# Effect of Lead and Copper on Photosynthetic Apparatus in Citrus (*Citrus aurantium* L.) Plants. The Role of Antioxidants in Oxidative Damage as a Response to Heavy Metal Stress

**DOI:** 10.3390/plants10010155

**Published:** 2021-01-14

**Authors:** Anastasia Giannakoula, Ioannis Therios, Christos Chatzissavvidis

**Affiliations:** 1 Laboratory of Plant Physiology, Department of Agriculture, International Hellenic University, 54700 Sindos, Greece; 2 Laboratory of Pomology, School of Agriculture, Aristotle University, 54124 Thessaloniki, Greece; therios@agro.auth.gr; 3 Department of Agricultural Development Democritus, University of Thrace, 68200 Orestiada, Greece; cchatz@agro.duth.gr

**Keywords:** antioxidant activity, chlorophyll, copper, hydrogen peroxide, lead, malondialdehyde

## Abstract

Photosynthetic changes and antioxidant activity to oxidative stress were evaluated in sour orange (*Citrus aurantium* L.) leaves subjected to lead (Pb), copper (Cu) and also Pb + Cu toxicity treatments, in order to elucidate the mechanisms involved in heavy metal tolerance. The simultaneous effect of Pb^−^ and Cu on growth, concentration of malondialdehyde (MDA), hydrogen peroxide (H_2_O_2_), chlorophylls, flavonoids, carotenoids, phenolics, chlorophyll fluorescence and photosynthetic parameters were examined in leaves of *Citrus aurantium* L. plants. Exogenous application of Pb and Cu resulted in an increase in leaf H_2_O_2_ and lipid peroxidation (MDA). Toxicity symptoms of both Pb and Cu treated plants were stunted growth and decreased pigments concentration. Furthermore, photosynthetic activity of treated plants exhibited a significant decline. The inhibition of growth in Pb and Cu-treated plants was accompanied by oxidative stress, as indicated by the enhanced lipid peroxidation and the high H_2_O_2_ concentration. Furthermore, antioxidants in citrus plants after exposure to high Pb and Cu concentrations were significantly increased compared to control and low Pb and Cu treatments. In conclusion, this study indicates that Pb and Cu promote lipid peroxidation, disrupt membrane integrity, reduces growth and photosynthesis and inhibit mineral nutrition. Considering the potential for adverse human health effects associated with high concentrations of Pb and Cu contained in edible parts of citrus plants the study signals that it is important to conduct further research into the accessibility and uptake of the tested heavy metals in the soil and whether they pose risks to humans.

## 1. Introduction

Toxic levels of heavy metals in the ecosystem is a global problem, threatening the survival of plants, wildlife and humans [1]. Elevated concentrations of both essential and non-essential heavy metals can lead to symptoms of toxicity and growth inhibition in most plants [2,3]. Since heavy metals are non-biodegradable, they accumulate in the environment and subsequently contaminate the food chain. Thus remediation of heavy metal pollution deserves due attention [4].

Copper (Cu) is an essential micronutrient for plants which is involved in many biochemical and physiological processes [5,6]. Cu compounds have been used not only for industrial activities but in agriculture as well. However, excessive Cu concentrations in plants can be toxic, as they lead to the induction of oxidative damage [7]. Since Cu compounds cannot be degraded by the soil microbiome, its toxicity is difficult to be eliminated, leading to great concern worldwide. High Cu concentrations in soil can be produced by Cu mining, discharge of domestic sewage and use of agricultural chemicals containing Cu. Cu is accumulated in plant tissues and is difficult to be scavenged [8].

Lead is not an essential element for plants although, it is still able to accumulate in different tissues and causes disturbances in physiological processes. Pb contamination of the orchards can be a result of mining activities, Pb containing materials and disposal of municipal sewage sludge [9]. Roots can take up significant quantities of Pb, however, its translocation to leaves is greatly restricted [10]. Therefore, a great amount of Pb remains in the roots [11] as it binds to cation exchange sites of the cell wall. Among heavy metals, Pb and Cu are potential pollutants accumulated in soils [12]. Plants developed various strategies to cope with Cu toxicity [13]. Pb is extremely toxic to all intermediates in the food chain [14] and to humans as well. The increase in heavy metal contaminants in agricultural soils depends on their rate of application and soil characteristics where the metals are applied [15]. The frequent use of Pb and Cu in orchards as insecticides or fungicides resulted in the pollution of the soil with high concentrations of these elements. Furthermore, pesticides, such as lead arsenate and copper sulfate, have been applied in orchards for more than fifty years and hence they cause food contamination.

Previous studies revealed the phytotoxic effects of high concentrations of heavy metals, including Pb, on growth, water potential and physiological, biochemical and molecular processes of plants [14,16]. A major consequence of abiotic and biotic stress in plants is the increased production of reactive oxygen species (ROS) [17]. The subsequent reduction of molecular oxygen to H_2_O yields the intermediates O^2−^, HO^•−^ and H_2_O_2_, which are potentially toxic, because they are relatively reactive, compared to O_2_ [18]. ROS may lead to unspecific oxidation of proteins and membrane lipids or may cause DNA damage. Therefore, tissues injured by oxidative stress generally contain increased concentrations of carbonylated proteins and malondialdehyde (MDA) [19]. Despite heavy metal toxicity, several plants are able to exclude, compartmentalize, accumulate or hyperaccumulate heavy metals and can also develop a wide range of adaptive strategies [20].

It is a well-known fact that the antioxidant defense system of plants is an adaptive strategy for the removal of toxic metals. The well-studied antioxidant systems consisting of low molecular weight antioxidants and specific enzymes. Recent studies have begun to highlight the potential role of flavonoids, phenylpropanoids, and phenolic acids as effective antioxidants [21]. Flavonoids are products of secondary metabolism with a vast array of functions, including antioxidative activity [22]. The functional diversity of flavonoids is due to their structural diversity, and to date more than 10,000 different molecules are known. This diversity endows flavonoids with many more biological functions, in addition to their roles as antioxidants in the plant [23]. Phenolic compounds, which could be also substrates for different peroxidases, are the first line of defence against Cu toxicity in an experiment with red cabbage [24]. Plant phenolic compounds such as flavonoids and lignin precursors have been recognized as beneficial antioxidants that can scavenge harmful ROS [7].

Among various metabolic processes, photosynthesis is one of the most significant physiological traits of plants. However, it has been reported to be negatively impacted by various heavy metals [25]. Photosynthetic inhibition during heavy metals stress is one of the primary actions in plants because they invariably affect photosynthetic apparatus and its functions, diminishing chlorophyll synthesis and inhibiting activities of the Calvin cycle either directly or indirectly, by inhibiting both light and dark reactions of photosynthesis [26,27,28]. Different plants exhibit diverse patterns of heavy metals accumulation which would influence biosynthesis of photosynthetic pigments. The accumulated heavy metals in plants may interact with the photosynthetic machinery which resulted in a wide variety of toxic effects, including photooxidative damage.

The objectives of the present work were to investigate the effects of Cu and Pb toxicity in *Citrus aurantium* L., an economically important Citrus rootstock and fruit tree dominant in the Citrus orchards in Greece. In addition, *Citrus aurantium* L. is among the species that have been used for medicinal purposes on account of the various bioactive compounds that it contains, such as phenolics, flavonoids, essential oils The effects of Cu and Pb toxicity on growth, photosynthetic pigments, H_2_O_2_, MDA, flavonoids, phenolics and chlorophyll fluorescence were studied. Cu and Pb were chosen as the toxic metals in this study because the surface horizons of many agricultural soils are rich in these elements due to their intensive use of agrochemicals and the application of manure and sludge, as organic fertilizers. In contrast, phytoremediation is the best solution to the metal toxicity problem. It is well known that phytoremediation is the use of plants to reduce the concentrations or toxic effects of heavy metals in the environment. Overall, higher plants have already shown their ability to fulfill all requisite conditions for the clean up of contaminated soils. This paper addresses various morphological, physiological and biochemical effects of Cu and Pb toxicity and strategies adapted by *Citrus aurantium* L. plants regarding Cu and Pb detoxification and plant tolerance.

## 2. Results

### 2.1. Effects of Copper and Lead on Plant Growth and Toxicity

The effects of heavy metals (Pb and Cu) were concentration-dependent. Hence, low Pb and Cu concentration increased 16% plant height compared to control (Table 1) however, combination of Pb + Cu significantly reduced plant weight.

Besides the decrease in plant height with Pb + Cu (500 μM), toxicity caused biochemical changes, with an increased concentration of H_2_O_2_, MDA and flavonoids by 73.8%, 20%, and 28.5%, respectively (Table 1). However, 800 μM of Cu and 800 μM Cu + Pb reduced plant height from 61% to 73% (Table 1). The toxic effects of the treatments 800 μM Pb, 800 μM Cu and 800 μM Cu + Pb reduced flavonoids to 24% of control values. The simultaneous addition of Pb and Cu resulted in peripheral chlorosis, bleaching, wilting and leaf necrosis after 60 days of treatment.

A typical toxic symptom in *Citrus aurantium* L. leaves due to high Pb and Cu concentration is leaf chlorosis. This symptom was not due to Ferric or Magnesium deficiency, but to toxic effects of Cu and Pb itself since our preliminary data (data are not shown) indicated that the increased level of Cu and Pb did not result in reduced concentrations of leaves on Mg and Fe.

### 2.2. Effect of Copper and Lead on Chlorophylls and Carotenoid Concentration

The increased concentrations of Pb and Cu exerted a considerable negative effect on chlorophylls and carotenoids (Table 2). The toxic effects of the treatments 800 μM Pb, 800 μM Cu and 800 μM Cu + Pb reduced chlorophylls 14–39.6% of control, carotenoids from 14–27% of control and phenols from 17–27%.

A concentration-dependent response of metal stress was observed on photosynthetic pigments. Hence, total chlorophyll concentration was significantly affected by Cu + Pb treatment (Table 2). The lowest Chl (a + b) was recorded at the high Cu + Pb level (40% of control) indicating severe stress and damage of photosynthetic apparatus whereas, marked maintenance in pigment content under lower simultaneous Cu + Pb (500 μM) was observed. With respect to chl a the effect of Pb and Cu was variable. Hence Pb (500 μM), Cu (500 μM) and Pb + Cu (500 μM) increased chla concentrations while the rest treatments reduced it. A typical toxicity symptom of *Citrus aurantium* L. leaves grown at high Pb and Cu concentration was leaf chlorosis. Chlorophyll concentration mainly varied between control and Cu + Pb stressed plants with the latter having less of chl and carotenoids. Furthermore, remarkable Cu + Pb induced changes in the ratio of chl to carotenoids were found and this ratio was equal to control with 800 μΜ Pb and 800 μΜ Cu, increased with 500 μΜ Cu + Pb and decreased with 500 μΜ Cu or 500 μΜ Pb. The effect of Pb and Cu on reducing total chl concentration in *Citrus aurantium* L. plant growing in a greenhouse was significant. In order to detect if the decrease in chl concentration affects photosynthetic efficiency, we measured the photochemical activity of PSII in dark-adapted leaves growing in Cu + Pb containing substrates, compared to control plants.

### 2.3. Effect of Copper and Lead on Chlorophyll Fluorescence

The maximum quantum efficiency of PSII photochemistry (Fv/Fm), as well as the efficiency of the water-splitting complex on the donor side of PSII (Fv/Fo), remarkably decreased depending on Pb or Cu concentration and their combination. The treatments 500 μM Cu and 500 μM Pb gave the same Fv/Fm and Fv/Fo values compared to control, while applications of 800 μM Pb, 800 μM Cu, 500 μΜ Cu + Pb and 800 μΜ Cu + Pb decreased significantly Fv/Fm and Fv/Fo values (Table 3).

The maximum quantum yield of charge separation in the PSII as measured by Fv/Fm reflected the changes of Fo or Fv. The Fv/Fm was significantly (*p* ≤ 0.05) affected by the Pb and Cu treatments.

The treatments with 500 μM Pb and 500 μM Cu, showed increased values compared to control by 10% and 15% respectively, while the values of 800 μM Pb, 800 μM Cu, 500 μM Cu + Pb and 800 μM Cu + Pb were decreased by 6%, 21%, 9% and 25% respectively compared to control.

### 2.4. Effect of Copper and Lead on H_2_O_2_ and Lipid Peroxidation

The plants growing in Pb + Cu solution significantly enhanced H_2_O_2_ content compared to control. Hence, the H_2_O_2_ concentration increased by 74% and 99% in 500 and 800 μM Pb + Cu containing substrates, respectively (Table 1). In all the treatments except 500 μM Pb (Table 1) the plants growing in Pb + Cu substrate significantly accumulated MDA, which is the end product of membrane fatty acid peroxidation compared to control. The MDA concentration in all the other treatments was 7% to 47% greater than control (Table 1). Treatment with high Pb + Cu concentrations (800 μM) initially resulted in a 47% increase in MDA concentration.

### 2.5. Effect of Copper and Lead on Total Flavonoids and Phenolics

The total flavonoid concentration was measured in methanolic extract of fresh leaf material. The results are shown in Table 1. The highest level of total flavonoid concentration (139%) compared to control was measured at 500μM Cu concentration (g CE x100 g^−1^dw) and the lowest one (77%) compared to control in the 800 μM Cu + Pb treatment. The values of 500 μM Pb, 500 μM Cu and, 500 μM Cu + Pb compared to control increased by 17%, 38%, 28%, in contrast to 800 μM Pb, 800 μM Cu and 800 μM Cu + Pb which showed 11%, 22% and 23% decrease respectively, compared to control. Additionally, our data indicated that Pb concentration influenced the total phenol concentration (Table 2). The highest phenol concentration was recorded at 500 μM Pb + Cu compared to control, whereas the lowest one at the highest Pb + Cu concentration.

### 2.6. Effect of Cu + Pb on Leaf Gas Exchange

**Figure 1:**
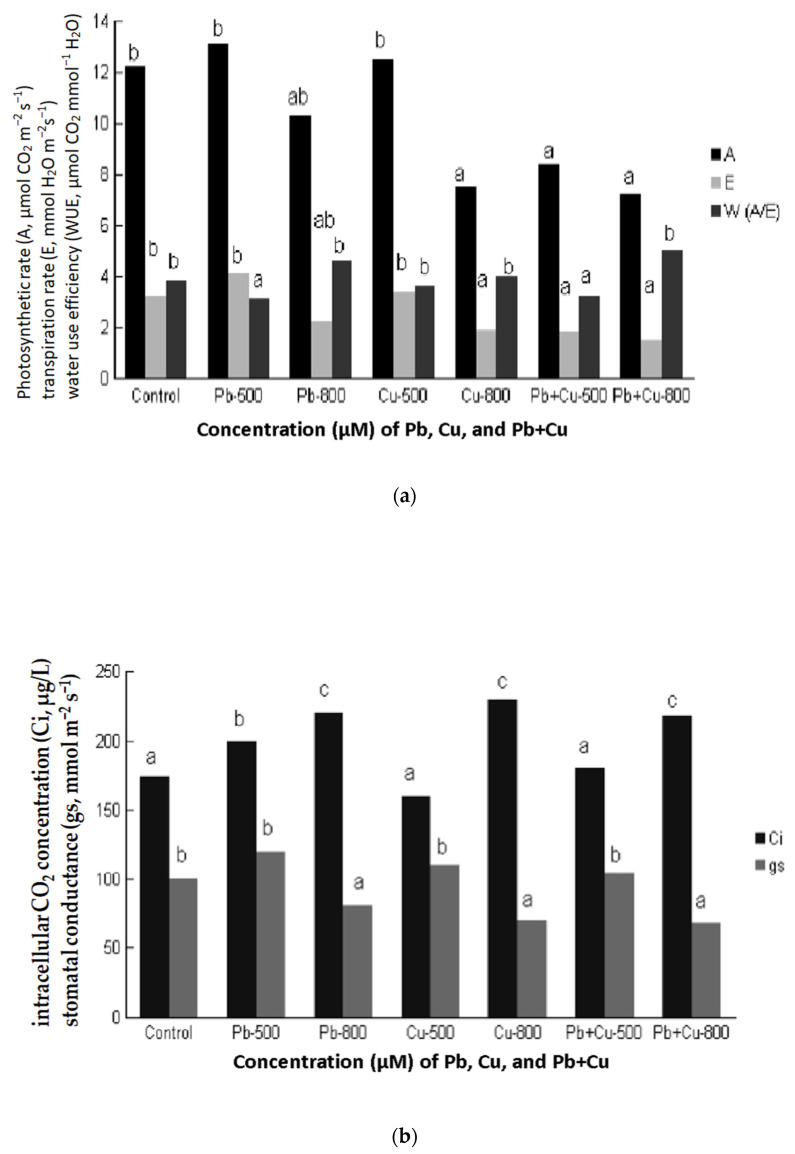
(**a**) Effects of Cu and Pb concentration (0 μΜ, 500 μΜ Pb, 800 μΜ Pb, 500 μΜ Cu, 800 μΜ Cu, 500 μΜ Cu + Pb, 800 μΜ Cu + Pb) in the nutrients solution on leaf net photosynthetic rate (A, μmol CO_2_ m*^−^*^2^ s*^−^*^1^); transpiration rate (E, mmol H_2_O m*^−^*^2^s*^−^*^1^) and water use efficiency (WUE, μmol CO_2_ mmol*^−^*^1^ H_2_O) of the youngest fully expanded leaf of *Citrus aurantium* L.plants. Each value is the mean of nine replicates. Means of the same parameter followed by different letters are significantly different. (*p* ≤ 0.05). (**b**) Effects of Cu and Pb concentration (0 μΜ, 500 μΜ Pb, 800 μΜ Pb, 500 μΜ Cu, 800 μΜ Cu, 500 μΜ Cu + Pb, 800 μΜ Cu + Pb) in the nutrients solution on intracellular CO_2_ concentration (Ci, μg/L), and stomatal conductance (gs, mmol m*^−^*^2^ s*^−^*^1^) of the youngest fully expanded leaf of *Citrus aurantium* L. plants. Each value is the mean of nine replicates. Means of the same parameter followed by different letters are significantly different (*p* ≤ 0.05).

Photosynthetic parameters are presented in Figure 1a,b. In Figure 1a. the parameters photosynthetic rate (A), transpiration rate (E) and water use efficiency (WUE) are presented. The treatments 500 μM Pb and 500 μM Cu did not exert any negative effect on photosynthetic rate while the treatments 800 μM Pb, 800 μM Cu, 500 μM Cu+ Pb and 800 μM Cu + Pb reduced significantly photosynthetic rate. The order of (A) decrease was: 800 μM Pb > 500 μM Cu + 500 μM Pb > 800 μM Cu ≥ 800 μM Cu + 800 μM Pb. The transpiration rate (Figure 1a) which was equal to control was recorded in the treatments 500 μM Cu and 500 μM Cu + 500 μM Pb.

The value of transpiration rate (E) in the treatments 800 μM Cu and 800 μM Cu + Pb was significantly decreased. Besides the treatments of 500 μM Cu and 500 μM Cu + Pb that were increased compared to control, all the other treatments were decreased. Finally, WUE (Figure 1a) was equal to control in the treatment 800 μM Pb, less than control in the treatments 500 μM Pb and 500 μM Cu + Pb and greater than control in the treatments 500 μM Cu, 800 μM Cu and 800 μM Cu + Pb. The intercellular CO_2_ concentration (Figure 1b) did not differ from control in the treatment 500 μM Cu, and alterations were recorded in all the others treatments following the order: 800 μM Cu > 800 μM Pb = 800 μM Cu + 800 μM Pb > 500 μM Pb = 500 μM Cu + 500 μM Pb > 500 μM Cu.

Stomatal conductance (gs) increased or decreased depending on the treatment. Hence, it was increased compared to control in the treatments 500 μM Cu, 500 μM Pb, 500 μM Cu + Pb and significantly decreased compared to control in the treatments 800 μM Pb, 800 μM Cu, 800 μM Cu + Pb.

## 3. Discussion

Plant growth inhibition under abiotic stresses creates a wide range of toxic effects in plants’ physiological and biochemical processes, with photosynthesis being the most sensitive one [17].

Heavy metals remaining in the soil play a significant role in the induction of plant stress [29]. Plants in order to resist this kind of stress developed a number of strategies including efflux pumps, sequestration in cells and in intracellular compartments, heavy metals binding into the cells and strong ligands production such as phytochelation. A majority of the heavy metal resistant plants prevent the accumulation of heavy metals inside the tissues [30]. Toxicity symptoms suggested that absorption of Pb and Cu by roots under different concentration levels of Pb and Cu in soil was significant. It is shown that Cu is more toxic to plants than Pb and that root growth is more sensitive to toxicity than shoot growth (data are not presented).

A common observation of previous Cu stress studies with Cu ecologically plausible concentrations [31] was that Cu usually does not affect the maximum dark-adapted quantum yield of PSII, measured as Fv/Fm [32]. In the present investigation, the increase in Fo and decrease in Fm under high Pb and Cu concentrations occurred concomitantly to a decrease in Fv/Fm. As previous researchers mentioned, two mechanisms at least, are involved in producing the changes in the fluorescence parameters under abiotic stress. One mechanism results in an increase in Fo, possibly due to the reduced plastoquinone acceptor (QA), being unable to be oxidized completely and the other is responsible for the decrease in Fm that indicates processes related to a decrease in the activity of the water-splitting enzyme complex [32].

Plant growth and development is a highly complex biological process and is required to be estimated in order to analyse the abiotic stress that has occurred Shoot growth was negatively affected by Cu and Pb toxicity. The decrease in shoot length in corn seedlings after Pb toxicity [33] was due to leakage of K ions from root cells. Probably the same mechanism exists in *Citrus aurantium* L. plants of our experiments. Other possible mechanisms are Pb accumulation in roots or the induction of an unknown signal in roots which is transmitted to shoots as a response to exposure to Pb or Cu [33]. Cu and Pb at a concentration of 800 μM inhibit chlorophyll and carotenoid biosynthesis and retard incorporation of these pigments into the photosynthetic machinery. Chlorophyll concentration is suggested to be very useful in vivo indicator of heavy metal toxicity for calculating the upper critical tissue concentrations. Accordingly, a recent study [34] has found that reduced Chla content accompanied by a significantly increased total phenolic content in leaves of *K. obovata* under high concentrations of heavy metal (Zn), enhances the heavy metal tolerance.

According to previous research [1], in *Oregano* plants after Cu stress, striking alterations in chloroplasts appeared. Hence, in control leaves, chloroplasts were of greater number and size, while in Cu treated leaves, chloroplasts had large plastoglobuli and dilation of the organelle’s limiting the double membrane. We propose that the same trend exists in the leaves of Cu + Pb treated *Citrus aurantium* L plants. This reduction in chlorophyll and carotenoid concentration in *Citrus aurantium* L. plants under Pb and Cu stress can be regarded as a specific plant response to metal stress, which resulted in chl degradation and inhibition of photosynthesis [35]. Additionally, the decrease in net photosynthesis follows, not only due to metal toxicity but also to reduced absorption of essential nutrients, which indirectly leads to chlorosis [36].

Photosynthesis is one of the most sensitive processes to Pb and Cu toxicity [37] and the effects are multi-dimensional affecting both in vivo as well in vitro photosynthetic CO_2_ fixation. It is widely accepted that PSII is very sensitive to several other types of environmental stresses. Cu is an essential micronutrient for higher plants with a major role in photosynthesis [27]. Cu is a constituent of the primary electron donor in the PSI of plants and of enzymes involved in the elimination of superoxide radicals. There is a strong linkage between Pb application and the decrease in photosynthesis in the whole plant and it is believed to result from stomatal closure rather than being a direct effect of Pb in the process of photosynthesis [4]. The process of photosynthesis (A) was adversely affected by Cu and Pb toxicity. Plants exposed to both metals showed a decline in photosynthetic rate which might have resulted from distorted chloroplast structure, restrained photosynthesis of chlorophyll and carotenoids, inhibition of Calvin cycle, as well as deficiency of CO_2_ due to stomatal closure [38]. Furthermore, Pb inhibits chlorophyll synthesis by causing inhibition of uptake of essential elements such as Mg or Fe by plants [39].

A high concentration of Pb in the nutrient substrate causes an imbalance of mineral nutrients in *Citrus aurantium* L. plants. According to previous workers [40] significant mineral imbalance as in the internal ratio of nutrients occur in plants under Pb toxicity, since Pb blocks the absorption of cations (K^+^, Ca^2+^, Mg^2+^, Mn^2+^, Zn^2+^, Cu^2+^ and Fe^3+^) in the root system. Pb also causes strong dissociation of the oxygen-evolving extrinsic polypeptide of PSII and displacement of Ca, Cl or Mn from the oxygen-evolving complex [41]. Accordingly, photosynthetic activity [42,43] is affected by many factors including stomatal conductance, which showed a significant reduction with the increased concentration of Cu + Pb. Cu toxicity causing reduction of PSII efficiency is associated with the thylakoid membranes of chloroplasts [44]. The reduction in the assimilation rate of *Citrus aurantium* L. plants treated with Pb and Cu was probably caused by stomatal closure, since Pb and Cu treated plants were accompanied by a lower gs as well as transpiration rate, especially those growing at higher levels of Pb and Cu (800 μM). The deleterious effect was most pronounced in all photosynthetic parameters after simultaneous Pb and Cu treatment at the highest level (800 μM). A decline in transpiration rate and water use efficiency occurs in *Citrus aurantium* L. plants growing under Pb exposure. Pb and Cu treatment causes growth retardation which results in a reduced leaf area, which is the main transpiration organ. Furthermore, Pb probably lowers the level of compounds that are associated with the maintenance of cell turgor and cell wall plasticity.

Decreased photosynthetic rates could be explained by the lower levels of chl(a + b). Nevertheless, the common link among different heavy metal stresses is the oxidative burst appearance. Heavy metals act by intercepting electrons from the photosynthetic electron transport chain, resulting in the production of toxic ROS. It is also well known that the toxic effect of heavy metals appears to be related to the production of ROS and the resulting unbalanced cellular redox status [45].

One of the phytotoxic effects of Pb appears to be the induction of oxidative stress in growing parts of *Citrus aurantium* L. due to enhanced production of reactive oxygen species (ROS) [7,46]. An important number of different ROS, including the superoxide anion (O^2−^), singlet oxygen (^1^O_2_), hydrogen peroxide (H_2_O_2_) and the hydroxyl radical (OH) are produced during the oxidative metabolism. Production of H_2_O_2_ in our study and its localization in leaf tissue contributed to elucidating the photoprotective mechanisms to Pb and Cu-induced oxidative stress conditions, confirming the conclusion [47]. Various abiotic stresses (such as heavy metal stress) lead to the overproduction of reactive oxygen species (ROS) in plants, which are highly reactive and toxic and cause damage to proteins, lipids and carbohydrates [48]. Pb induces the production of ROS within plants and such production depends on the intensity of the stress. Pb induces lipid peroxidation and decreases the level of fatty acids [49]. In addition, oxidative stress has often been described as a result of Cu stress, as a consequence of inhibition of the photosynthetic light reaction [6]. Lower H_2_O_2_ accumulation is correlated with environmental stress tolerance [50]. The production of H_2_O_2_ described in several studies leads to a better understanding of plant responses to stress. The plants growing in Pb and Cu substrate significantly enhanced H_2_O_2_ concentration compared to control. Such an observation is in agreement with an earlier study reporting increased H_2_O_2_ concentration in plant roots under Pb-stress in *Hypnum plumaeforme* [51], and pea plants [7]. Nevertheless, H_2_O_2_ accumulation is another ROS that is implicated in enhanced lipid peroxidation and membrane damage-causing cell death [52]. Treatment with increased Pb and Cu concentrations (800 μM) initially resulted in a significant increase in MDA concentration These observations are in conformity with an earlier observation [53]. Additionally, other workers [54] reported MDA accumulation in *Nasturtium officinale* in response to Pb.

Furthermore, an enhanced content of antioxidants was observed in the leaves of *Citrus aurantium* L. plants growing under Pb and Cu treatment. Our data suggest that these strategies are involved with protection against oxidative damage, thus protection of the photosynthetic machinery. Previous investigations [55] have also confirmed that plants that produce high amounts of phenolic compounds as a response to the heavy metal stress could be good candidates for phytoremediation.

Therefore, phytoremediation as a promising technology for the removal of contaminated cultivated lands can ensure the consumption of agricultural products such as fruits, which are an important component of the human diet.

## 4. Material and Methods

### 4.1. Plant Material and Culture

One-year-old *Citrus aurantium* L. plants were grown with different concentrations of Cu and Pb in a greenhouse at the Aristotle University farm in Thessaloniki, Greece, (40°34′35″ Ν 22°57′19″ Ε), during 2013. *Citrus aurantium* L. plants were grown in plastic pots containing 1.2 L of 1:1 mixture of sand and perlite. The experimental plants were irrigated every two days with a modified Hoagland nutrient solution [56]. All macronutrients and micronutrients were supplied at half strength. Additionally, the nutrient solution contained Cu(SO_4_)_2_ and Pb(NO_3_)_2_. The experiment included 7 treatments: control, 500 and 800 μM Cu(SO_4_)_2_ 500 and 800 μM Pb(NO_3_)_2_ 500 μM Cu(SO_4_)_2_ + 500 μM Pb(NO_3_)_2_. 800 μMCu(SO_4_)_2_ + 800 μM Pb(NO_3_)_2_. For each treatment, 9 pots (replications) were used. Control plants were treated only with the modified Hoagland nutrient solution free of Pb and of additional Cu except its quantity as a micronutrient in the Hoagland solution.

### 4.2. Chlorophyll and Carotenoid Estimation

The third from the top fully expanded leaf was sampled for analysis of chlorophylls and carotenoids, on the 60th day from the initiation of Pb and Cu stress. For estimation of photosynthetic pigments, fresh leaf blade material (0.1 g) was placed in 25 mL glass test tubes and 15 mL of 96% (*v*/*v*) ethanol was added to each tube. The tubes with the plant material were incubated in a water bath at a temperature of 79.8 °C until complete discoloration of samples, after about three to four hours. The absorbance of chlorophylls a and b was measured at 665 and 649 nm, respectively. Total chlorophyll was determined [57]. Carotenoids concentration was estimated on a vis spectrophotometer [58,59].

### 4.3. In Vivo Chlorophyll Fluorescence Measurements

In vivo chlorophyll fluorescence was measured on the upper leaf surface of the third from the top fully expanded leaf, using a Plant Analyser (PEA, Hansatech Ltd., King’s Lynn, Norfolk, UK) and Fv/Fm, Fv/Fo ratios were determined [60]. Measurements were conducted at 23 °C, on intact leaves of four replicate plants from the nine treatments.

### 4.4. Photosynthesis Measurements

Gas exchange was measured on the third, from the top fully expanded leaf with IRGA Li-6400 portable photosynthesis meter (LiCor, Inc. Lincoln, NE, USA). Calculations of net photosynthetic rate (A), transpiration rate (E), water use efficiency (WUE = A/E), intercellular CO_2_ concentration (Ci) and stomatal conductance (g_s_) from gas exchange measurements were conducted [61]. Leaf temperature was about 25 °C, the relative humidity (RH) was 60–70%, CO_2_ concentration was 400 μL L^−1^, and light intensity was 1000 μmoLm^−2^ s^−1^.

### 4.5. MDA Content

The level of lipid peroxidation of *Citrus aurantium* L. leaves at the end of the experiment was measured and the MDA content was determined by reaction with 2-thiobarbituric acid (TBA) [62]. Fresh leaf blade tissue, 0.1 g homogenised by adding 0.5 mL of 0.1% (*w*/*v*) trichloroacetic acid (TCA). The homogenate was centrifuged at 15,000× *g* and 4 °C for 10 min. From the supernatant 0.5 mL was mixed with 1.5 mL of 0.5% TBA diluted in 20% TCA. Incubation follows at 95 °C for 25 min. The reaction stops by incubating on an ice bath. Afterward, the tubes were centrifuged at 10,000× *g* and 4 °C for 10 min and the absorbance of the supernatant was read at 532 and 600 nm. The value for the non-specific absorption at 600 nm was substrated from the value at 532 nm. The concentration of MDA was calculated using Lambert–Beer’s law using the MDA extinction coefficient of 155 mM^−1^ cm^−1^ [63]. Results are presented as μmol MDA g^−1^ FW.

### 4.6. Determination of H_2_O_2_ Concentration

For determination of H_2_O_2_ concentration in leaves of *Citrus aurantium* L. plants after 60 days of Pb + Cu treatment, leaf extraction was carried out according to [64]. Hydrogen peroxide was measured spectrophotometrically after reaction with KI. The reaction mixture consisted of 0.5 mL leaf extract with 0.1% trichloroacetic acid (TCA), 0.5 mL of 100 mM of K-phosphate buffer and 2 mL reagent (1 M KI in fresh double-distilled H_2_O). The blank consisted of 0.1% TCA. The reaction was developed for 1 h in darkness. The absorbance of the solution was measured at 410 nm and H_2_O_2_ concentration was calculated using a standard curve ranging from 0.1 to 1 mM. H_2_O_2_ content and was expressed as nmol g*^−^*^1^ FW.

### 4.7. Sample Preparation for Antioxidants

The third from the top leaf was sampled and 0.1 g (FW), was divided into small pieces, put in a mortar and was homogenised with 1mL of 80% methanol. The centrifugation followed at 12,000× *g* and 4 °C for 20 min. The supernatants were collected and stored at −80 °C.

### 4.8. Evaluation of Total Flavonoid Content

The total flavonoid content was measured using a colorimetric method [65] with minor modifications. The extraction solution used was the same as that for phenolic compounds. Aliquots (0.5 mL) of samples or standard solutions were pipetted into 15 mL polypropylene conical tubes containing 2 mL of double-distilled H_2_O and mixed with 0.15 mL of 5% NaNO_2_ After 5 min 0.15 mL of 10% AlCl_3_ 6H_2_O solution was added. The mixture was allowed to stand for another 5 min and then 1 mL of the 1 M NaOH was added. The reaction solution was mixed well and after 15 min the absorbance A_415_ was determined.

The standard curve was prepared using different concentrations of catechin. The aluminium ion (Al^3+^) is reacted with flavonoids in the sample to form the stable flavonoid-Al^3+^ complex which has a yellow colour and intensity proportional to flavonoid concentration. The test should be performed at least in triplicate and flavonoid content was expressed in g of catechin equivalents (CE) per 100 g of DW of the sample.

### 4.9. Determination of Phenolics

Total phenolics content was determined by the Folin–Ciocalteu method [66] with some modifications. The powdered sample (500 mg) was extracted with 50 mL of 80% methanol for 30 min on a hot plate. The extract was filtered through a filter paper into a 50 mL volumetric flask and the volume was completed using the same solvent. The solution of the reaction consists of 2400 μL Folin–Ciocalteu (1:10 *v*/*v*), 80% (*v*/*v*) methanolic extract (100 μL) and nanopure water (500 μL). These chemicals were combined in tubes and then mixed via magnetic stirring. The mixture was allowed to react for 3 min and then 2 mL of Na_2_CO_3_ (7.5% *w*/*v*) solution was added and mixed well. The solution was incubated at 37 °C for 5 min. The phenolic compounds in the sample are oxidised using the Folin–Ciocalteu reagent. The reagent is a mixture of phosphomolybdic and phosphotungstic acids that are reduced by the oxidation of phenolic compounds and the blue colour produced by the oxides has a maximum absorption at 760 nm, which is proportional to the total phenolic concentration.

The tubes were left to be cooled at room temperature (23 °C). The absorbance was measured at 760 nm using a spectrophotometer (Prim, SECOMAM, France) and the results were expressed in gallic acid equivalents (GAE; mg/100 g fresh mass) using a gallic acid standard curve.

### 4.10. Statistical Analyses

All the data were processed by the statistical package SAS (version 9.0, Destiny Corporation, Rocky Hill, CT, USA, 2014). Values reported are the means of four from the nine replicates. Data were tested for significant differences of (*p* ≤ 0.05) using one way ANOVA.

## 5. Conclusions

In conclusion, this study indicates that Lead and Copper provide a wide range of adverse effects on physiological processes. It is also well documented that ROS production under heavy metal stress promoted lipid peroxidation of membranes and caused disruption of their integrity (increased MDA and H_2_O_2_). Pb and Cu toxicity leads to inhibition of antioxidant activity, disturbed mineral nutrition, water imbalance and disorders of the photosynthetic machinery. The process of photosynthesis is adversely affected by Cu + Pb toxicity. Furthermore, Pb and Cu toxicity decreased plant height and increased the concentration of MDA, H_2_O_2_ and flavonoids. The main symptom of Pb and Cu toxicity is chlorosis, wilting and leaf necrosis. The increased concentration of Pb and Cu exerted a considerable negative effect on pigments, chlorophyll fluorescence and photosynthetic parameters. Although this study will probably enlighten the mechanism of the biochemical basis of Pb and Cu tolerance in plants, further studies are needed to specify the biochemical parameters associated with antioxidant defence system and tolerance to Pb and Cu in *Citrus aurantium L.* plants.

Further study on the effectiveness of citrus plants as bioindicators in the contaminated orchards is necessary to be carried out in order to assess the potential of adaptation mechanisms according to these heavy metals that were examined.

## Figures and Tables

**Table 1:** Effects of Cu(SO_4_)_2_ and Pb(NO_3_)_2_ on plant height (cm), H_2_O_2_ (mol/g FW) malondialdehyde (MDA) (nmol/g FW) and flavonoids (g CE./100 g DW) of *Citrus aurantium* L. plants. The values of plant height, H_2_O_2_, MDA and flavonoids are the mean of nine replications (plants); quality characteristics are the mean of five different measurements, from the nine replications. Means in the same column followed by different letters are significantly different (*p* ≤ 0.05).

Treatments	Concentration μM	Plant Height cm	H_2_O_2_ mol/g FW	MDA nmol/g FW	Flavonoids g CE./100 g DW
Control	0	50 ^c^	10.31 ^a^	25.4 ^a^	128.4 ^c^
Pb	500 μM	58.0 ^d^	10.23 ^a^	23.3 ^a^	151.6 ^d^
Pb	800 μM	49.1 ^c^	16.93 ^b^	29.5 ^b^	115.1 ^b^
Cu	500 μM	52.2 ^c^	15.41 ^b^	27.3 ^b^	178.8 ^e^
Cu	800 μM	40.1 ^b^	18.23 ^b^	33.3 ^a^	101.7 ^a^
Cu + Pb	500 μM	42.1 ^b^	17.92 ^c^	30.5 ^b^	165.1 ^e^
Cu + Pb	800 μM	31.2 ^a^	20.41 ^c^	37.3 ^c^	98.8 ^a^

**Table 2:** Effects of Cu(SO_4_)_2_ and Pb(NO_3_)_2_ on chlorophylls (Chl (a + b), Chl a), carotenoids and Phenolics (GAE; mg/100 g FW) of *Citrus aurantium* L. (Nine replications) plants. Quality characteristics are the mean of five measurements. Means in the same column followed by different letters are significantly different (*p* ≤ 0.05).

Treatments	Concentration μM	Chl(a + b) mg g^−1^ DW	Chla mg g^−1^ DW	Carotenoids mg g^−1^ FW	Phenolics GAE mg/100 g FW
Control	0	11.7 ^c^	7.60 ^c^	8.30 ^c^	223.4 ^c^
Pb	500 μM	12.6 ^d^	8.84 ^d^	10.3 ^d^	281.2 ^d^
Pb	800 μM	10.1 ^b^	6.75 ^b^	7.2 ^b^	187.1 ^b^
Cu	500 μM	13.2 ^d^	9.85 ^d^	10.9 ^d^	284.5 ^d^
Cu	800 μM	8.6 ^a^	6.02 ^a^	6.3 ^a^	171.2 ^a^
Cu + Pb	500 μM	12.9 ^d^	8.71 ^d^	7.2 ^b^	287.1 ^d^
Cu + Pb	800 μM	7.2 ^a^	5.63 ^a^	6.1 ^a^	164.5 ^a^

**Table 3:** Effects of Cu(SO_4_)_2_ and Pb(NO_3_)_2_ on chlorophyll fluorescence parameters (Fo, Fm, Fv/Fm, Fv/Fo) of *Citrus aurantium* L. (Nine replications, plants). Quality characteristics are the mean of five measurements. Means in the same column followed by different letters are significantly different (*p* ≤ 0.05).

Treatments	Concentration μM	Fo	Fm	Fv/Fm	Fv/Fo
Control	0	437 ^c^	2200 ^c^	0.81 ^a^	4.0 ^a^
Pb	500 μM	430 ^d^	2538 ^b^	0.83 ^a^	4.9 ^a^
Pb	800 μM	481 ^c^	2012 ^c^	0.78 ^b^	3.6 ^b^
Cu	500 μM	379 ^d^	2409 ^bc^	0.85 ^a^	4.2 ^a^
Cu	800 μM	1006 ^a^	3383 ^a^	0.64 ^d^	2.4 ^c^
Cu + Pb	500 μM	509 ^d^	1912 ^d^	0.74 ^c^	2.7 ^c^
Cu + Pb	800 μM	1107 ^a^	3651 ^a^	0.61 ^d^	2.2 ^c^
